# Molecular evidence for the transovarial passage of *Babesia gibsoni* in *Haemaphysalis hystricis* (Acari: Ixodidae) ticks from Taiwan: a novel vector for canine babesiosis

**DOI:** 10.1186/s13071-018-2722-y

**Published:** 2018-03-20

**Authors:** Frans Jongejan, Bi-Ling Su, Hsiang-Ju Yang, Laura Berger, Judith Bevers, Pin-Chen Liu, Jou-Chien Fang, Ya-Wen Cheng, Charlotte Kraakman, Nadine Plaxton

**Affiliations:** 1 Utrecht Centre for Tick-borne Diseases (UCTD), FAO Reference Centre for Ticks and Tick-borne Diseases, Faculty of Veterinary Medicine, Utrecht University, Yalelaan 1, 3584 CL Utrecht, The Netherlands; 2 Vectors and Vector-borne Diseases Research Programme, Department of Veterinary Tropical Diseases, Faculty of Veterinary Science, University of Pretoria, Private Bag X04, Onderstepoort, 0110 South Africa; 3 Institute of Veterinary Clinical Sciences, School of Veterinary Medicine, National Taiwan University, No.1, Sec. 4 Roosevelt Road, Taipei, 106 Taiwan

**Keywords:** Taiwan, *Babesia gibsoni*, *B. vogeli*, *Haemaphysalis hystricis*, *Rhipicephalus sanguineus*, Transovarial transmission

## Abstract

**Background:**

*Babesia gibsoni* is the predominant tick-borne protozoan blood parasite affecting dogs throughout the Oriental region. *Babesia gibsoni* is transmitted by *Haemaphysalis longicornis*, whereas a similar role has been suggested for *Rhipicephalus sanguineus*. *Haemaphysalis longicornis* does not occur in Taiwan, but *R. sanguineus* is widely distributed on dogs. However, clinical cases of babesiosis are mainly restricted to the northern part of the island. The discrepancy between tick distribution and clinical cases stimulated us to investigate the tick species distribution on dogs in northern Taiwan, with the aim to identify the local vector for canine babesiosis.

**Methods:**

Ticks were collected from stray dogs or free ranging pet dogs in northern Taiwan between 2015 and 2017 and, after identification, were tested for the presence of tick-borne *Babesia* parasites using PCR and reverse line blot (RLB) hybridisation. Moreover, engorged ticks collected from the dogs were incubated at 28 °C to allow them to oviposit. Their subsequent larval progeny was also examined by PCR/RLB.

**Results:**

A total of 1085 ticks collected from 144 stray dogs at different residential areas consisted of 5 different species: *H. hystricis* (*n* = 435), *R. sanguineus* (*n* = 582), *R. haemaphysaloides* (*n* = 43), *Amblyomma testudinarium* (*n* = 14) and *Ixodes ovatus* (*n* = 11) were identified. *Babesia gibsoni* DNA was detected in *H. hystricis* females (10.3%), males (7.0%) and in 2.6% of the nymphs. One *R. sanguineus* female and one *A. testudinarium* female tick also carried *B. gibsoni* DNA. DNA of *B. gibsoni* was demonstrated in 11 out of 68 (16.2%) batches of larval ticks derived from engorged *H. hystricus* ticks only. *Babesia vogeli* DNA was detected only in *R. sanguineus* females (2.6%) and males (2.4%). DNA of *B. vogeli* was detected in 13 out of 95 (13.7%) batches of larval ticks derived from engorged *R.sanguineus* females.

**Conclusions:**

*Babesia gibsoni* DNA was detected in the larval progeny of *H. hystricis* ticks only, whereas *B. vogeli* was restricted to the larvae of *R. sanguineus*. This provides evidence for transovarial passage of *B. gibsoni* in *H. hystricis* and evidence that this tick does act as the local vector for this parasite on dogs in northern Taiwan where most cases of babesiosis are reported. The vectorial capacity of *R. sanguineus* for babesiosis is probably restricted to the transmission of *B. vogeli* only.

## Background

Babesiosis is an important tick-borne haemoprotozoan disease, which occurs worldwide in a broad range of domestic and wild animal species as well as in humans [1]. Babesiosis in dogs is characterised by a wide range of clinical manifestations from subclinical to severe disease characterized by hemolytic anaemia and disseminated intravascular coagulation [2]. Although differences between breeds of dogs play a role, the main reason for the diverse clinical presentation is the involvement of different *Babesia* species, which traditionally are divided into two groups according to the relative size of the piroplasm stage within the canine erythrocyte [3]. *Babesia canis*, *Babesia vogeli* and *Babesia rossi* are large *Babesia* species, whereas *Babesia gibsoni* and “Babesia vulpes” are characterized by small intra-erythrocytic piroplasms [4–6]. In addition to morphology, significant differences have been reported with respect to their clinical presentation and cross-immunity in dogs [3]. Moreover, there are distinct differences in geographical distribution, serological profile, molecular phylogeny and, last but not least, in their association with particular tick vectors [3, 7, 8].

*Babesia canis* is found in the Palaearctic region coinciding with the distribution of its vector tick *Dermacentor reticulatus*. This tick has been linked to the transmission of *B. canis* in several original field and laboratory studies conducted in France, Germany and the Netherlands [3, 9–11]. Moreover, Shortt [11] in a historical paper worked with *Babesia rossi* in *Haemaphysalis leachi* (now *H. elliptica*) from South Africa, and not with *Babesia canis*.

*Babesia vogeli* has a worldwide distribution coinciding with the cosmopolitan vector tick, *Rhipicephalus sanguineus* (*sensu lato*). Experimental evidence that *R. sanguineus* can transmit *B. vogeli* has been published [2, 3, 7].

*Babesia gibsoni* is endemic in Asia, where it is transmitted by *Haemaphysalis longicornis* ticks between dogs [12]. Outside Asia, *B. gibsoni* infections are often associated with Pit Bull Terriers and other fighting dogs, whereby it has been hypothesised that an asexual strain of *B. gibsoni* is maintained in the fighting dog population without genetic recombination in the vector tick [4].

In Taiwan, *B. gibsoni* is the predominant protozoan blood parasite affecting the health of domestic and stray dogs. A five-year retrospective survey among dogs presented at the National Taiwan University between 2008 and 2012 confirmed that *B. gibsoni* is the most important infectious pathogen causing severe anaemia [13]. Since Taiwan is free of *Haemaphysalis longicornis*, *R. sanguineus* has been suggested to act as a vector for *B. gibsoni* [14]. However, *R. sanguineus* is widely distributed on dogs, but clinical cases of babesiosis are mainly restricted to the northern part of the island. Here, the tick species distribution on dogs in northern Taiwan was investigated with the aim to identify the local tick vector for canine babesiosis.

## Methods

### Tick collection and breeding

**Fig. 1:**
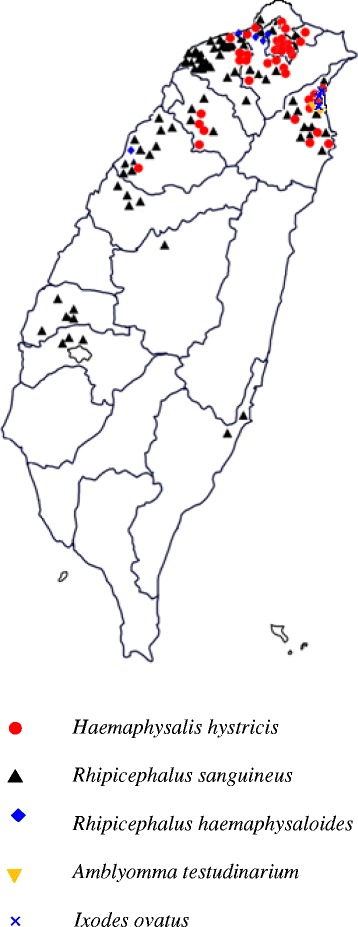
Map of Taiwan with the locations where the different tick species were found on dogs

A total of 144 dogs were sampled for ticks at different residential locations for three consecutive years (2015–2017) (Fig. 1). Ticks were removed using forceps and transported in ventilated tubes to the laboratory for identification. Unfed ticks were stored in 70% ethanol, whereas engorged females were incubated at 28 °C and 85% relative humidity in the dark for production of eggs and hatching of subsequent larvae. All ticks were tested by PCR, including the larval progenies of the engorged females.

### PCR amplification and reverse line blot hybridisation

Ticks were disrupted in 2 ml microcentrifuge tubes containing extraction buffer and stainless steel beads using a Tissuelyser LT (Qiagen Benelux BV, Venlo, The Netherlands). DNA was extracted from the triturated ticks using a DNA extraction kit (Fisher Scientific, Landsmeer, The Netherlands) according to the manufacturer’s instructions. Extracted DNA was either directly used or stored at -20 °C. After DNA extraction, DNA was PCR amplified and tested by using reverse line blot hybridisation (RLB) first applied for differential diagnosis and detection of tick-borne *Babesia* species by Gubbels [15].

For PCR, the primer pair RLB-F2 (5′-GAC ACA GGG AGG TAG TGA CAA G-3′) and RLB-R2 (5′-biotin-CTA AGA ATT TCA CCT CTG ACA GT-3′) [16, 17]) (Thermo Fisher Scientific, Breda, The Netherlands) was used to amplify the V4 variable region of the *18S* rRNA gene of *Babesia* and *Theileria* species. The length of the PCR amplicon was 460 bp. PCR was performed in a total volume of 25 μl, containing 5 μl of a 5× Phire PCR buffer, 0.5 μl of 2 mM dNTP Mixture, 0.5 μM each primer, 0.125 μl of 5 U/μl Phire Hot Start II polymerase (Thermo Fisher Scientific), 2.5 μl of extracted genomic DNA, and double distilled water.

As positive controls, genomic DNA from *B. gibsoni* as well as from *Babesia bovis* was used. No template was used as negative control. Strict standard operating procedures were followed to prevent contamination, which included separate rooms for each PCR reaction step and a unidirectional workflow. PCR hoods were equipped with an anti-microbial UV lamp operated at intervals to sterilise the work environment and positive displacement pipettes with barrier tips were used to prevent carry-over from one sample to the next. Finally, the entire work surface was sterilized with a bleach dilution between subsequent PCR assays.

Oligonucleotide probes containing an N-terminal N-(trifluoracetamidohexyl-cyanoethyl, N, N-diisopropyl phosphoramidite [TFA])-C6 amino linker was also synthesised by Thermo Fisher Scientific. In addition to *B. gibsoni* and *B. vogeli*, eight other *Babesia* species were targeted by these probes (*Babesia canis*, *Babesia rossi*, *Babesia venatorum*, *Babesia ovis*, *Babesia caballi*, *Babesia bovis* and *Babesia bigemina*)(16). Catch-all *Theileria/Babesia* probes were included to capture possible unknown species or variants of species (17).

RLB hybridisation was conducted as previously described [15]. Briefly, a Biodyne C membrane was activated at room temperature using 16% (wt/wv) 1-ethyl-3-(3-dimethyl-aminopropyl) carbodiimide (EDAC) (Carl Roth GmbH, Karlsruhe, Germany) for 10 min, after which the oligonucleotide probes were covalently linked to the membrane in 0.5 M NaHCO_3_ in a mini-blotter. The membrane was inactivated in 100 mM NaOH after washing in 2× SSPE/0.1% SDS at 60 °C and then stored in 20 mM EDTA, pH 8.0. For the assays, 10 μl of PCR product was added to 150 μl of 2× SSPE/0.1%SDS after denaturing at 100 °C for 10 min, followed by immediate cooling on ice. Denatured PCR products were then hybridised to a pre-prepared Biodyne C membrane at 42 °C for 60 min. The membrane was subsequently washed twice in preheated 2× SSPE/0.5% SDS at 50 °C for 10 min, incubated for 30 min at 42 °C in 2× SSPE/ 0.5% SDS with 2.5 μl of streptavidin-POD conjugate (Roche Diagnostic, Mannhein, Germany), washed twice in preheated 2× SSPE/ 0.5% SDS at 42 °C for 10 min, and finally washed twice in 2× SSPE for 5 min at room temperature. Hybridization detection was performed using chemiluminescence.

## Results

In total, 1085 ticks were collected from 144 dogs at different residential locations mainly in Northern Taiwan (Fig. 1). Collections were carried out during the spring and summer activity period of the ticks for three consecutive years starting in May 2015 until the end of September 2017. The species composition of the adult ticks recovered from the dogs revealed the presence of five different species. *Rhipicephalus sanguineus* was the predominant species followed by *H. hystricis*, whereas small numbers of *Rhipicephalus haemaphysaloides*, *Amblyomma testudinarium* and *Ixodes ovatus* were also identified (Table 1).

**Table 1:** Species composition of ticks identified on stray dogs in the North of Taiwan with their respective *Babesia gibsoni* infection

	*Babesia gibsoni-*positive / No. of ticks tested (%)				
	Male	Female	Nymph	Larva	Total
*Haemaphysalis hystricis*	4/57 (7.0)	23/223 (10.3)	2/77 (2.6)	11/68 (16.2)	40/435 (9.2)
*Rhipicephalus sanguineus*	0/127 (0)	1/343 (0.3)	0/17 (0.0)	0/95 (0)	1/582 (0.2)
*Rhipicephalus haemaphysaloides*	0/1 (0)	0/42 (0)	0/0 (0.0)	0/0 (0)	0/43 (0)
*Amblyomma testudinarium*	0/0 (0)	1/14 (7.1)	0/0 (0.0)	0/0 (0)	1/14 (7.1)
*Ixodes ovatus*	0/0 (0)	0/11 (0)	0/0 (0.0)	0/0 (0)	0/11 (0)

**Fig. 2:**
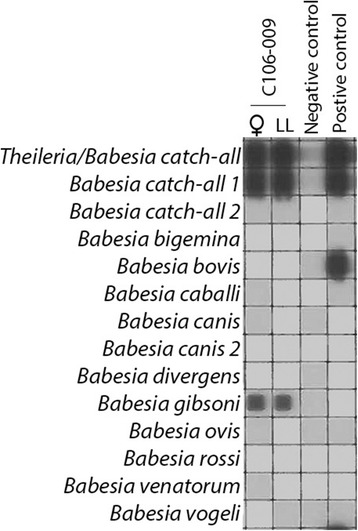
Reverse line blot wherein transovarial passage of *Babesia gibsoni* in *Haemaphysalis hystricis* ticks is demonstrated

**Fig. 3:**
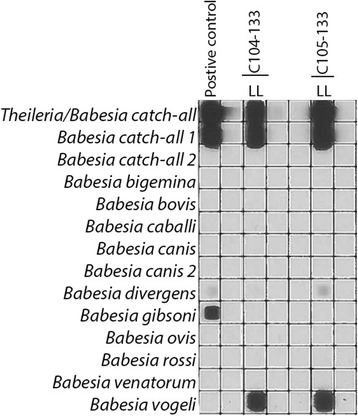
Reverse line blot wherein transovarial passage of *Babesia vogeli* in *Rhipicephalus sanguineus* ticks is demonstrated

*Haemaphysalis hystricis* (*n* = 435), *Rhipicephalus sanguineus* (*n* = 582), *Rhipicephalus haemaphysaloides* (*n* = 43), *Amblyomma testudinarium* (*n* = 14) and *Ixodes ovatus* (*n* = 11) were tested by PCR/RLB (Tables 1, 2). Engorged females of *R. sanguineus* (*n* = 95) and *H. hystricis* (*n* = 68) were allowed to oviposit eggs, and their resulting larval progeny was tested, together with all of the unfed or partially fed tick stages (*n* = 922). *Babesia gibsoni* DNA was detected in *H. hystricis* females (10.3%), males (7.0%) and in 2.6% of the nymphal ticks (Table 1). One *R. sanguineus* female and one *A. testudinarium* female tick were also infected with *B. gibsoni*. There was *B. gibsoni* DNA in 11 out of 68 (16.2%) batches of larval ticks produced by engorged *H. hystricis* ticks recovered from the dogs, suggesting transovarial passage (Table 1, Fig. 2). *Babesia vogeli* DNA was detected in *R. sanguineus* females (2.6%) and males (2.4%). There was *B. vogeli* DNA in 13 out of 95 (13.7%) batches of larval ticks produced by engorged *R. sanguineus* ticks only (Table 2, Fig. 3).

**Table 2:** Species composition of ticks identified on stray dogs in the North of Taiwan with their respective *Babesia vogeli* infection

	*Babesia vogeli-*positive / No. of ticks tested (%)				
	Male	Female	Nymph	Larva	Total
*Haemaphysalis hystricis*	0/57 (0)	0/223 (0)	0/77 (0)	0/68 (0)	0/435 (0)
*Rhipicephalus sanguineus*	3/127 (2.4)	9/343 (2.6)	0/17 (0)	13/95 (13.7)	21/582 (3.6)
*Rhipicephalus haemaphysaloides*	0/1 (0)	0/42 (0)	0/0 (0)	0/0 (0)	0/43 (0)
*Amblyomma testudinarium*	0/0 (0)	1/14 (0)	0/0 (0)	0/0 (0)	0/14 (0)
*Ixodes ovatus*	0/0 (0)	0/11 (0)	0/0 (0)	0/0 (0)	0/11 (0)

## Discussion

It is possible that ticks may ingest *Babesia* with their blood meal and become PCR positive without being able to transmit the parasite. To differentiate between PCR-positive ticks and those that do act as vectors for infectious disease agents, our approach was to allow engorged female ticks to oviposit their eggs and subsequently test their larval progeny. If the parasite is detected within the next generation of ticks, this will provide a strong indication that subsequent transmission can occur.

In this study, it was demonstrated that transovarial passage of *B. gibsoni* occurred in 16.2% of the larval batches of *H. hystricis* (Table 1, Fig. 2). Likewise, there was the transovarial passage of *B. vogeli* in 13.7% of larval batches of *R. sanguineus* ticks (Table 2, Fig. 3). This is strong evidence for a role of *H. hystricis* as a local vector of canine babesiosis, which has not been reported before. Transovarial passage of *B. vogeli* in *R. sanguineu*s confirms its involvement in the transmission canine babesiosis.

*Haemaphysalis hystricis* appears to have a distribution restricted to the northern part of Taiwan (Fig. 1). However, very recently the tick was reported on dogs (*n* = 9) together with many more *R. sanguineus* (*n* = 306) in Nantou County, which is in the central part of Taiwan (Fig. 1) [18]. The finding that *H. hystricis* can act as a vector of canine babesiosis may have regional relevance, since this tick is not restricted in its distribution to Taiwan [19]. *Haemaphysalis hystricis* Supino, 1897 is the East Asian mountain haemaphysalid, found in India, Sri Lanka, Vietnam, Myanmar, China, Japan, Indonesia, Laos, Thailand, as well as in Taiwan [20]. The tick is prominently listed by Robbins in his synonymic checklist of ticks of Taiwan [21]. *Haemaphysalis hystricis* appears to be a tick of low to intermediate altitudes from sea level to approximately 7000 ft elevation, which in these latitudes are usually subtropical and available data suggest that this is a forest-inhabiting species. Concerning preferred hosts, the tick has a fairly wide host range from medium size to large carnivores, deer, wild boar, but is also found on domestic dogs and occasionally attacks humans [20]. Other domestic animals and birds are not infested by *H. hystricis* [22].

In addition to the vector role of *H. hystricis* for babesiosis in dogs, a few other pathogens vectored by this tick are worth mentioning. A stercorarian trypanosome species have been isolated from naturally infected *H. hystricis* collected in Kagoshima Prefecture in Japan [23]. Also, in Japan, *H. hystricus* was among ticks collected from dogs in Ehime Prefecture, Shikoku, an endemic area for Japanese spotted fever, suggesting dogs as a host of the vector ticks for Japanese spotted fever control [24]. Finally, a novel *Borrelia* species was isolated from *H. hystricis* collected from wildlife in an Orang Asli settlement in Selangor, Malaysia [25]. In Taiwan, *B. gibsoni* appears the only infectious disease agent thus far associated with *H. hystricis*.

In the Oriental region, *H. hystricis* may act as a vector of canine babesiosis in areas outside the distributional range of *H. longicornis* [19, 22]. The role of *H. longicornis* as a vector for *B. gibsoni* was initially demonstrated by the detection of developmental stages in gut epithelium, hemolymph as well as salivary glands of this tick [26–28]. Subsequently, the role of *H. longicornis* as principle vector of canine babesiosis caused by *B. gibsoni* has been confirmed by many molecular studies, for instance by those conducted in Japan [29, 30]. *Babesia gibsoni* can now accurately be quantified in tick tissues using a modified TaqMan probe-based qPCR system that targets parasite DNA in combination with the SYBR Green dye system [31].

On the other species of ticks found in this survey, *R. sanguineus* was the predominant species. *Babesia vogeli* infections that were found in this tick have been previously reported in Taiwan [32]. Also, this tick has been phylogenetically characterized and was found to be affiliated with the tropical lineage group of the *R. sanguineus* (*sensu lato*) group [33]. Interestingly, in a similar series of morphological papers as conducted with *H. longicornis*, Higuchi and co-workers reported the development of *B. gibsoni* in the midgut and salivary glands of larval *R. sanguineus* [34, 35], as well as in the midgut of the nymphal stage of this tick [36]. Although transovarial passage of *B. gibsoni* was demonstrated with a series of developmental stages culminating in sporozoites in the salivary glands, experimental transmission to dogs was not carried out [26].

Recently, *B. gibsoni* was for the first time identified in nymphs, male and females of *R. sanguineus* ticks also collected from dogs also in Taiwan [14]. Nearly 1200 ticks collected from veterinary practitioners and pet clinics were examined for *Babesia* infection, and the infection rates of nymphs, males and female ticks were 2.42%, 0.98% and 1.97%, respectively. Sequence and phylogenetic analysis revealed that these *Babesia* spp. were *B. gibsoni* and *B. vogeli*. More specifically, two *Babesia* strains (GenBank: KU884667 and KU884668) from partial-engorged nymphs were *B. gibsoni,* and the remaining 16 *Babesia* strains (GenBank: KU884669–KU884684) from various stages of the ticks were *B. vogeli* [14]. In our study, one out of 343 female *R. sanguineus* was found infected with *B. gibsoni.*

Further evidence for a possible vector role of *R. sanguineus* for *B. gibsoni* has recently been presented in an epidemiological survey of ticks and tick-borne pathogens in pet dogs conducted in China [37]. Ticks (*n* = 1550) were collected from 562 dogs presented at 122 veterinary clinics from 20 major cities in south-eastern China. Among 617 ticks tested by PCR, 8/453 (1.77%) of *R. sanguineus* and 5/91 (5.49%) of *H. longicornis* were infected with *B. gibsoni* [37].

There were low numbers of *R. haemaphysaloides, A. testudinarium* and *I. ovatus* ticks identified on the dogs in this study. The numbers collected were not sufficient to determine whether they could play any role in the epidemiology of canine babesiosis. Interestingly, in our study, none of the *R. haemaphysaloides* ticks was found infected, whereas 1/73 (1.37%) of *R. haemaphysaloides* ticks collected in the recent study in China was infected with *B. gibsoni* [37]. *Rhipicephalus haemaphysaloides* was previously reported from cattle in Taiwan [38] and on small mammals in different parts of Taiwan [39]. This tick was the predominant tick species and contained a high diversity of *Rickettsia* spp. [39]. Recently, experimental transmission of *Babesia microti* by *R. haemaphysaloides* was demonstrated [40].

In our study, one *A.testudinarium* female contained *B. gibsoni* DNA. Other pathogen relationships have not been reported for this tick, although it was recently genetically analysed after it was identified as a human biting tick species. Finally, *Ixodes ovatus* has previously been collected from dogs in Taiwan [41] and cats [42]. First detection and molecular identification of *Borrelia garinii* in Taiwan were from an *I. ovatus* tick recovered from a stray cat [42]. Our specimens (*n* = 11) were all negative for *Babesia* infections.

Finally, if one considers the global distribution of *B. gibsoni*, some epidemiological findings need to be mentioned. *Babesia gibsoni* is widespread throughout the Oriental region, for instance in China [43]. However, cases of canine babesiosis caused by *B. gibsoni* have been reported outside the distributional range of its main vector tick, *H. longicornis*, incriminating other ticks for its transmission. The first evidence of *B. gibsoni* (Asian genotype) in dogs in western Europe was reported in two American Pit Bull Terriers [44]. Subsequently, cases have been reported in Croatia, Italy, Serbia, Slovakia, Spain and the UK [2]. A recent large study among pit bull-type fighting dogs in USA revealed that *B. gibsoni* was the predominant infectious disease agent with 39% of 269 dogs positive [45]. The infection can be transmitted by blood or saliva through bites, and this type of clonal expansion may take place without a sexual cycle through ticks. Furthermore, in Brazil, *B. vogeli* [46] as well as *B. gibsoni* [47] have been reported and transmitted by *R. sanguineus* [48]. However, the global distribution of *R. sanguineus* (*sensu lato*) does not coincide with the distribution of *B. gibsoni.* Maybe there are subpopulations within this diverse phylogenetic group that is unable to transmit. Although *R. sanguineus* has been incriminated, its vectorial ability has not been demonstrated under laboratory conditions [2]. This needs further investigation.

Ultimately, experimental transmission of *B. gibsoni* by known and putative vector ticks is required in controlled clinical trials, wherein *B. gibsoni*-positive dogs are simultaneously infested with different tick species for parasite acquisition and separately tested for their capacity to transmit to susceptible dogs. This will provide the final proof of their vectorial capacity.

Finally, it will be interesting to investigate the natural hosts of *B. gibsoni* in Taiwan as recently determined for the causative agent of canine babesiosis in South Africa [49]. Small wildlife mammals such as civet, ferret badger and mongoose, which do occur in Northern Taiwan are possible targets for such an investigation since they are frequently encountered in the same area as where some of the stray dogs were sampled for this study.

## Conclusion

The transovarial passage of *B. gibsoni* in *H. hystricis* provides evidence that this tick does act as the local vector for this parasite on dogs, whereas the vectorial capacity of *R. sanguineus* is probably restricted to *B. vogeli* only.

## Abbreviations

qPCR: Quantitative real-time polymerase chain reaction
RLB: Reverse line blot
SDS: Sodiumdodecylsulfate
SSPE: Sodium chloride-sodium phosphate-EDTA
Streptavidine-POD: Streptavidine-peroxidase
